# Integrated Analysis of mRNA and Non-coding RNA Transcriptome in Pepper (*Capsicum chinense*) Hybrid at Seedling and Flowering Stages

**DOI:** 10.3389/fgene.2021.685788

**Published:** 2021-08-20

**Authors:** Huang-ying Shu, He Zhou, Hai-ling Mu, Shu-hua Wu, Yi-li Jiang, Zhuang Yang, Yuan-yuan Hao, Jie Zhu, Wen-long Bao, Shan-han Cheng, Guo-peng Zhu, Zhi-wei Wang

**Affiliations:** Key Laboratory for Quality Regulation of Tropical Horticultural Crops of Hainan Province, College of Horticulture, Hainan University, Haikou, China

**Keywords:** pepper, heterosis, RNA-seq, lncRNA, miRNA, transcription factor

## Abstract

Pepper is an important vegetable in the world. In this work, mRNA and ncRNA transcriptome profiles were applied to understand the heterosis effect on the alteration in the gene expression at the seedling and flowering stages between the hybrid and its parents in *Capsicum chinense*. Our phenotypic data indicated that the hybrid has dominance in leaf area, plant scope, plant height, and fruit-related traits. Kyoto Encyclopedia of Genes and Genomes analysis showed that nine members of the plant hormone signal transduction pathway were upregulated in the seedling and flowering stages of the hybrid, which was supported by weighted gene coexpression network analysis and that *BC332_23046* (auxin response factor 8), *BC332_18317* (auxin-responsive protein IAA20), *BC332_13398* (ethylene-responsive transcription factor), and *BC332_27606* (ethylene-responsive transcription factor WIN1) were candidate hub genes, suggesting the important potential role of the plant hormone signal transduction in pepper heterosis. Furthermore, some transcription factor families, including bHLH, MYB, and HSF were greatly over-dominant. We also identified 2,525 long ncRNAs (lncRNAs), 47 micro RNAs (miRNAs), and 71 circle RNAs (circRNAs) in the hybrid. In particular, downregulation of miR156, miR169, and miR369 in the hybrid suggested their relationship with pepper growth vigor. Moreover, we constructed some lncRNA–miRNA–mRNA regulatory networks that showed a multi-dimension to understand the ncRNA relationship with heterosis. These results will provide guidance for a better understanding of the molecular mechanism involved in pepper heterosis.

## Introduction

Heterosis is a fundamental biological phenomenon characterized by a superior agricultural performance that enhances plant biomass, growth rates, seed yield, and stress tolerance, compared with their inbred parents. Hybrids have been comprehensively applied to agriculture, to increase yield and quality. Three classical hypotheses have been proposed to explain the genetic control of heterosis: dominance (Xiao et al., 1995; Garcia et al., 2008; Li et al., 2014), overdominance (East, 1936; Larièpe et al., 2012; Zhou et al., 2012), and epistasis (Powers, 1944). Subsequently, comprehensive research suggested that quantities of superior alleles were related to heterosis (Yu et al., 1997; Melchinger et al., 2007). Tremendous progress has been made in characterizing the genetic mechanisms of plant heterosis, including *Arabidopsis* (Groszmann et al., 2014; Yang et al., 2017), rice (Li et al., 2016; Shao et al., 2019), maize (Ding et al., 2014; Hu et al., 2016), oilseed (Shen et al., 2017), and tomato (Krieger et al., 2010). For example, studies suggest that DNA methylation (Kawanabe et al., 2016; Lauss et al., 2017) and non-coding RNA (ncRNA) are linked to heterosis (Ng et al., 2012; Li et al., 2014). These data provide important hints for studying the heterosis of other plants. However, the molecular mechanism of heterosis still remains to be further deciphered (Govindaraju, 2019).

Pepper (*Capsicum* L.) is a genus of the *Solanaceae* family, which is an important vegetable and ornamental plant. Pepper spread all over the world because of its superior adaptability to diverse agroclimatic regions (Qin et al., 2014). The global pepper planting area was about ∼1.99 million hectares with a production of ∼36.77 million tons (FAO, 2018). Moreover, pepper plays an important role in pharmaceuticals, cosmetics, and pigmentation (Kothari et al., 2010). Pepper hybrids show strong heterosis and have been broadly used in commercial production. However, the molecular mechanism of heterosis in pepper is poorly understood, which seriously impedes the efficient breeding of pepper hybrids.

The application of new biotechnologies, including next-generation sequencing on the *Capsicum chinense* (one of the five cultivated species of the *Capsicum* genus), helped to explore the heterosis of pepper at the genomic level (Kim et al., 2014, 2017; Qin et al., 2014). The weighted gene coexpression network analysis (WGCNA) is a promising tool to reveal coexpressed gene networks or modules that are based on transcriptome data, which combines phenotypic features with these modules to detect the key genes in the networks by WGCNA (Langfelder and Horvath, 2008). This procedure has been extensively used to identify the module key genes of plant horticulture traits, but fewer studies have applied WGCNA to illustrate the gene networks underlying pepper heterosis.

In the present study, we aimed to provide comprehensive information to study heterosis and hybrid breeding by using non-directional sequencing, strand-specific RNA sequencing, and small RNA analysis. We implemented transcriptomic analysis at the pepper seedling stage (S-stage) and flowering stage (F-stage) to explore the candidate genes related to heterosis. Through WGCNA, we identified some possible genes that play a role in the networks. Moreover, we detected the differential transcriptome containing lncRNA, circRNA, and miRNA among two parents and their F_1_ hybrid at the S-stage of *C. chinense*. Overall, these data will be helpful for further illustration of the molecular mechanism of heterosis and open new ways to study heterosis in pepper.

## Materials and Methods

### Plant Materials and Morphological Observation

Pepper plants (*C. chinense*) of HNCc16, HNCc22, and their hybrid HNCy01 with HNCc22 as the maternal parent were planted in the growth room with 16 h light (3,500 Lx) at 26°C followed by 8 h dark at 20°C. The plant height (PH), stem diameter, and leaf area (LA) were measured (30 days at the S-stage and 70 days at the F-stage). The LA was calculated using ImageJ (v1.51). Leaves were frozen immediately in liquid nitrogen after harvest and stored at –80°C for RNA extraction (triplicate biological duplications for each sample).

### RNA Extraction and RNA-Seq

The total RNA of each sample was isolated with Trizol reagents under the manufacturer’s instruction (Thermo Fisher Scientific, Shanghai, China). Eighteen non-directional libraries were produced using NEBNext^®^ Ultra^TM^ RNA Library Prep Kit for Illumina^®^ (NEB, United States) and sequenced on Illumina Novaseq platform. Nine strand-specific libraries were produced by rRNA-depleted RNA by NEBNext^®^ Ultra^TM^ Directional RNA Library Prep Kit for Illumina^®^ with the dUTP second-strand marking (NEB, Ipswich, MA, United States) and used for generating mRNA, lncRNA, and circRNA data. Moreover, nine small RNA libraries were constructed using NEBNext^®^ Multiplex Small RNA Library Prep Kit for Illumina^®^ (NEB, Ipswich, MA, United States) and used for generating miRNA data. The cleaned Illumina reads were aligned to the pepper reference genome^1^ using HISAT2 (v.2.0.4) (Kim et al., 2015). All raw data were deposited in the National Center for Biotechnology Information Sequence Read Archive with accession numbers: PRJNA694379 for non-directional sequencing library and PRJNA655561 for strand-specific RNA sequencing library and small RNA library. The samples MRS30, RS30, and YS30 were from the S-stage, and MRF60, RF60, and YF60 were from the F-stage. The sample names MRs, Rs, and Ys in the database are equivalent to HNCy01, HNCc22, and HNCc16 in this paper^2^.

### Differential Expression Analysis

Fragments per kilo-base of exon per million fragments mapped (FPKM) of lncRNAs and mRNAs in each sample was calculated by StringTie (v2.1.1) (Pertea et al., 2016). Expression levels of miRNAs and circRNAs were estimated by transcript per million (TPM) (Zhou et al., 2010). Differentially expressed genes (DEGs), DE lncRNAs, DE miRNAs, and DE circRNAs were analyzed by the DESeq2 (v1.16.1) (Love et al., 2014). | Log2(foldchange)| > 1 and *p*-adjust < 0.05 were set as the threshold for significantly differential expression by default. The DEGs were classified into five major expression patterns based on the gene expression level: additive, high-parental dominance, low-parental dominance, under-dominance, and over-dominance (Li et al., 2014). The coexpression network visualization of differential ncRNAs was conducted by the Cytoscape (v3.8.0) software^3^. All of the Venn diagrams were drawn by Jvenn^4^ (Bardou et al., 2014), and heat maps were drawn by R package (pheatmap).

### Gene Ontology and Kyoto Encyclopedia of Genes and Genomes Enrichment Analysis

Gene Ontology (GO) enrichment of DEGs was analyzed by GOseq (Young et al., 2010). Kyoto Encyclopedia of Genes and Genomes (KEGG) is a public database resource for annotating the biological system (Kanehisa et al., 2008). Using KOBAS software, we identified the enrichment of the target DE candidate genes, DE lncRNAs, and DE miRNAs in KEGG pathways (Mao et al., 2005).

### Transcription Factors Analysis

Transcription factors (TFs) were analyzed by iTALK (v1.2) software^5^ (Pérez-Rodríguez et al., 2010).

### Analysis of WGCNA Gene Coexpression Network

The weighted gene coexpression network analysis was used to identify genes for network construction, gene cluster, and visualization (Langfelder and Horvath, 2008). The correlation of pepper phenotypic traits was analyzed by R package corPvalueStudent function. The gene correlation and soft thresholding power analysis were based on Pearson correlation matrix. In WGCNA networks, a power of 10 was chosen (model fitting index *R*^2^ = 0.8). The gene dendrogram was used for module detection, and dynamic tree cutting method was used (minModuleSize and mergeCutHeight value were set as 100 and 0.25, respectively). The phenotype data were imported into WGCNA package, and the correlation between phenotype and gene module was calculated using default settings.

## Results

### Morphological Comparison of F_1_ Hybrid and Its Parents

**FIGURE 1:**
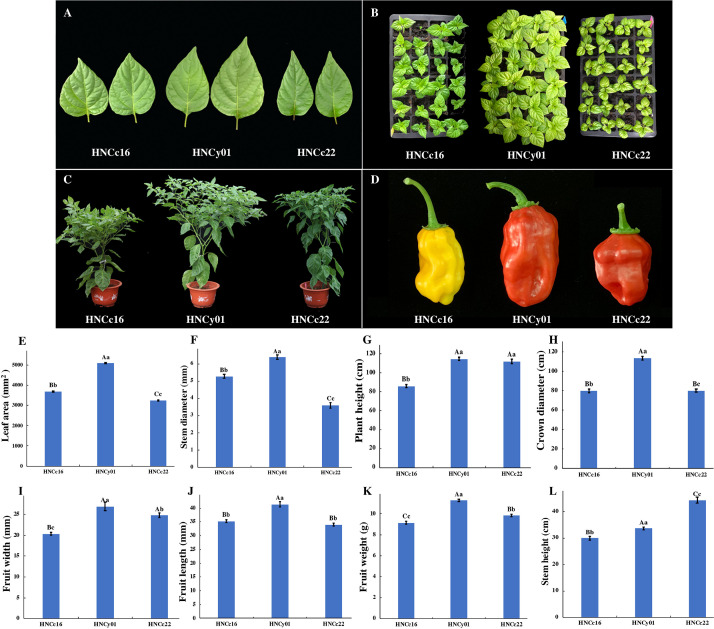
The growth of the F_1_ hybrid and its pepper parents at the S-stage and F-stage. **(A)** Leaves from F_1_, HNCc22, and HNCc16 at the S-stage. **(B)** Plants of the F_1_ and its parents at 35 days after germination. **(C)** Plants of the hybrid and its parents at F-stage of 70 days after germination. **(D)** The fruit of the hybrid and its parents. **(E,F)** Plant LA and stem diameter of the hybrid and parents at the S-stage. **(G–L)** Plant height, crown diameter, fruit width, fruit length, fruit weight, and stem height of hybrid and its parents at the F-stage. Different letters above the columns of bars indicate significant differences at *p*-value less than 0.05 (lowercase letters) or less than 0.01 (capital letters).

We collected the pepper hybrid and its parents at S-stage and F-stage for RNA sequencing analysis (RNA-seq). These two periods play a vital role in the growth and heterosis interpretation of pepper. Moreover, both F_1_ hybrid HNCy01 and maternal line HNCc22 were more vigorous at these two phases, and the hybrid showed higher PH than the paternal line HNCc16. At S-stage, the hybrid LA was larger than the parents, and the diameter of the stem of the hybrid was similar to that of the thick stem parent (HNCc16). The hybrid crown diameter and PH have a greater advantage than its parents at F-stage. Moreover, fruit diameter, fruit length (FL), and fruit weight (FW) also showed strong hybrid vigor (Figure 1).

### Sequencing and Mapping Reads to the Pepper Reference Genome

We performed RNA sequencing of the three genotypes at the S-stage and F-stage and constructed 18 libraries. A total of 83.72 million raw reads were generated from the three genotypes at S-stage and F-stage through RNA-seq on Illumina HiSeq 2500 platform. The paired-end sequences with low-quality reads were filtered out. Finally, 80.92 million clean reads were obtained. On average, GC content was 42.42%, and Q30 was 93.5%. Interestingly, about 91.02% of reads were mapped to the *C. chinense* reference genome, and 86.71% were aligned to unique positions. Among these reads, 51.35–67.77% of reads were distributed to exonic regions, 27.25–43.84% to intergenic, and 4.49–7.94% to intronic regions, respectively (Supplementary Table 1).

To comprehensively investigate how the hybrid influences the transcript profile, we also constructed nine strand-specific RNA libraries (used for acquiring mRNAs, lncRNAs, and circRNAs) and nine small RNA libraries. For strand-specific RNA sequencing, the raw data size of each sample ranges from 87.73 to 158.25 million reads, obtaining 83.56–150.49 million clean reads. For small RNA sequencing, the numbers of raw reads were distributed from 13.02 to 20.36 million, including 11.59–19.72 million clean reads. Thus, at least 84.9% of strand-specific clean data in each sample were aligned with the reference genome, and over 76.07% of small RNAs from each sample were mapped to the reference genome (Supplementary Table 2).

### Differential Gene Expression Analysis

**FIGURE 2:**
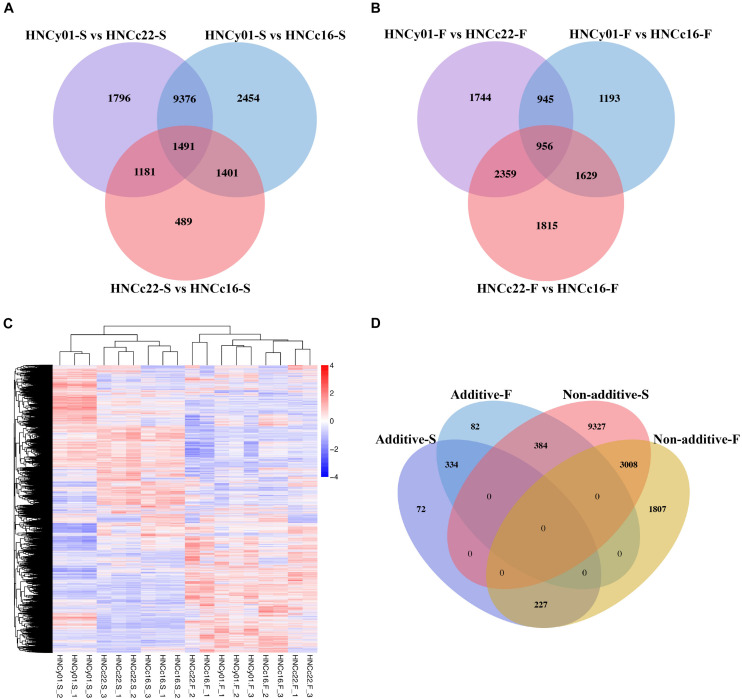
Differential expression between the hybrid and its parents at the two development stages. **(A)** S-stage and **(B)** F-stage. **(C)** Heatmap and cluster analysis of expression level of DEGs. **(D)** Comparison of additive and non-additive genes in the hybrid at the S-stage and F-stage.

At the S-stage, 28,566 DEGs were identified between hybrid HNCy01 and the parental line, and 4,562 DEGs were discovered between the parent HNCc22 and HNCc16 with a similar number of upregulated DEGs and downregulated DEGs. At the F-stage, there were 10,727 among HNCy01 and its parents and 6,759 between inbred, respectively (Supplementary Figure 1). Through a Venn diagram, we identified 2,672 and 3,315 DEGs between the hybrid HNCy01vs HNCc22 and HNCc22vs HNCc16 at S-stage and F-stage, respectively, with 2,892 and 2,585 DEGs between HNCy01vs HNCc16 and HNCc22vs HNCc16 at both stages. In addition, 1,491 and 956 DEGs were identified among three genotype combinations at the two stages (Figures 2A,B). Moreover, hierarchy analysis showed that different samples at the same development stages clustered together (Figure 2C).

By studying differential gene expression among the hybrid and its parents in two development stages, we found that a total of 633 and 800 DEGs showed additive effects in the S-stage and F-stage among hybrid HNCy01 and its parents, respectively. Whereas 12,719 and 5,042 DEGs at S-stage and F-stage displayed non-additivity effects, the non-additive genes were further divided into four patterns: high-parent dominance, low-parent dominance, over-dominance, and under-dominance (Table 1). Moreover, research on the comparison of gene expression patterns displayed that 3,008 non-additive genes were retained in both stages. In the F-stage, 384 genes showed an additive pattern, but in the S-stage, they showed a non-additive pattern. Besides, 334 additive genes were maintained in these two stages, and a total of 227 genes displayed non-additive expression patterns at the F-stage but additive patterns at the S-stage (Figure 2D). These results indicated that the non-additive expression genes played an important role in the different development stages.

**TABLE 1:** Differentially expressed genes between the hybrid and its parents at the seedling and flowering stages.

Expression patterns		S-stage	F-stage
Total		13,352	5,842
Additive		633 (4.74%)	800 (13.69%)
Non-additive		12,719 (95.26%)	5,042 (86.31)
High-parental dominance	ELD-F	721	1,286
	ELD-M	818	987
Low-parental dominance	ELD-F	448	1,047
	ELD-M	565	627
Over-dominance		4,869	550
Under-dominance		5,298	545

### Enrichment Analysis of Gene Ontology and Kyoto Encyclopedia of Genes and Genomes

**FIGURE 3:**
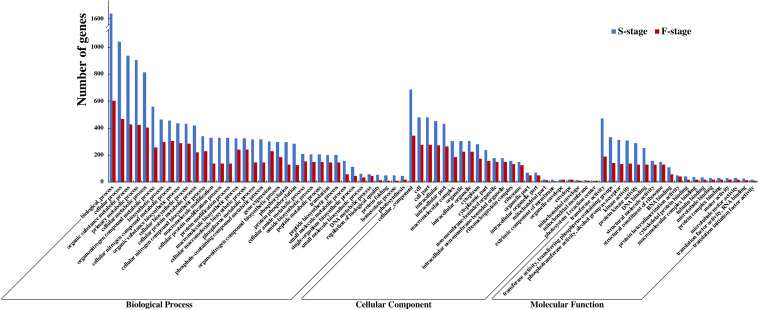
Comparison of Gene Ontology (GO) classification of differentially expressed genes (DEGs) at the seedling and flowering stages.

To delve into an overall insight in the molecular and biological functions of DEGs between the hybrid and its parents, we identified 12,719 and 5,042 non-additively expressed genes at S-stage and F-stage, respectively. Among HNCy01 and its parents at these two stages, the biology process, cellular process, organic substance metabolic process, and primary metabolic process were enriched most significantly in the biological process category. In the cellular component category, the cellular component, cell, cell part, and photosystem were enriched. In the molecular function category, the transferase activity, transferring phosphorus-containing groups, phosphotransferase activity, alcohol group as acceptor, and kinase activity were significantly abundant (Figure 3). Furthermore, we inspected that serval terms exhibited over-dominance expression patterns at both development stages (Table 2).

**TABLE 2:** Significant Gene Ontology (GO) terms of over-dominance at the seedling and flowering stages.

GO accession	Description	Term type	*Q*-value
GO:0016310	Phosphorylation	biological_process	1.9034E-09
GO:0006468	Protein phosphorylation	biological_process	2.7427E-09
GO:0006793	Phosphorus metabolic process	biological_process	5.7423E-07
GO:0006796	Phosphate-containing compound metabolic process	biological_process	5.7423E-07
GO:0016773	Phosphotransferase activity, alcohol group as acceptor	molecular_function	5.6832E-10
GO:0016301	Kinase activity	molecular_function	8.1118E-10
GO:0004672	Protein kinase activity	molecular_function	1.5464E-09
GO:0016772	Transferase activity, transferring phosphorus-containing groups	molecular_function	1.9132E-07
GO:0016740	Transferase activity	molecular_function	0.0001406

**FIGURE 4:**
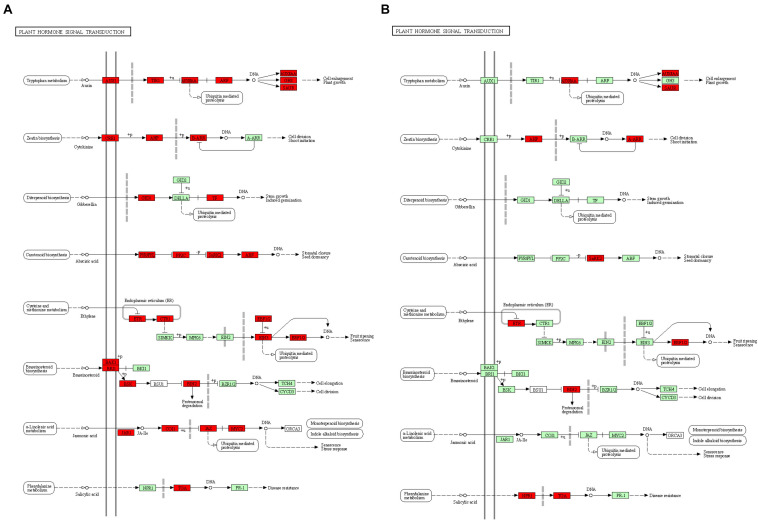
The “plant hormone signal transduction” pathway enriched by Kyoto Encyclopaedia of Genes and Genomes (KEGG) analysis. **(A)** At S-stage. **(B)** At F-stage. Upregulated KEGG Orthology (KO) nodes are marked with red.

Kyoto Encyclopedia of Genes and Genomes analysis of DEGs at the two development stages displayed non-additional enrichment pathways, including ribosome biogenesis in eukaryotes, proteasomes, plant hormone signal transduction, ribosomes, photosynthesis-antenna proteins, and photosynthesis at the S-stage. However, at the F-stage, protein processing in the endoplasmic reticulum, photosynthesis, photosynthesis-antenna proteins, plant hormone signal transduction, and carotenoid biosynthesis were significantly enriched (Supplementary Figure 2). Interestingly, we found that plant hormone signal transduction, photosynthesis, and photosynthesis-antenna proteins were high-parental dominant at the S-stage and over-dominant at the F-stage. Particularly, the plant hormone signal transduction pathways were extremely enriched, where *BC332_21619*, *BC332_18317*, *BC332_12434*, *BC332_25121*, and *novel.2052* were over-dominant in both developmental stages. These results strongly suggest the contribution to the pathway for the growth vigor in the F_1_ hybrid (Figure 4 and Supplementary Tables 3, 4).

### Analysis of Coexpressed Gene Networks and Candidate Genes

**FIGURE 5:**
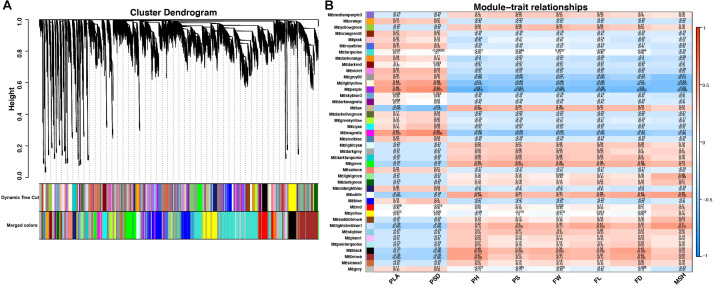
Cluster dendrogram and network heatmap of coexpressed genes. **(A)** Hierarchical cluster of 42 modules coexpressed genes. **(B)** Module–trait relationship. Each row represents a module eigengene, and each column presents a trait. Each module includes the corresponding correlation and *p*-value. PLA, plant leaf area; PSD, plant stem diameter; PH, plant height; PS, plant scope; FL, fruit length; FD, fruit diameter; FW, fruit weight; MSH, main stem height.

To reveal the transcriptional regulation of pepper heterosis based on RNA-seq data among the hybrid and inbred at two development stages, WGCNA was used to identify 42 modules of coexpressed genes to reveal the correlation between genes and traits indexes. These modules were represented by different colors and displayed with a heatmap (Figure 5A and Supplementary Figure 3).

**FIGURE 6:**
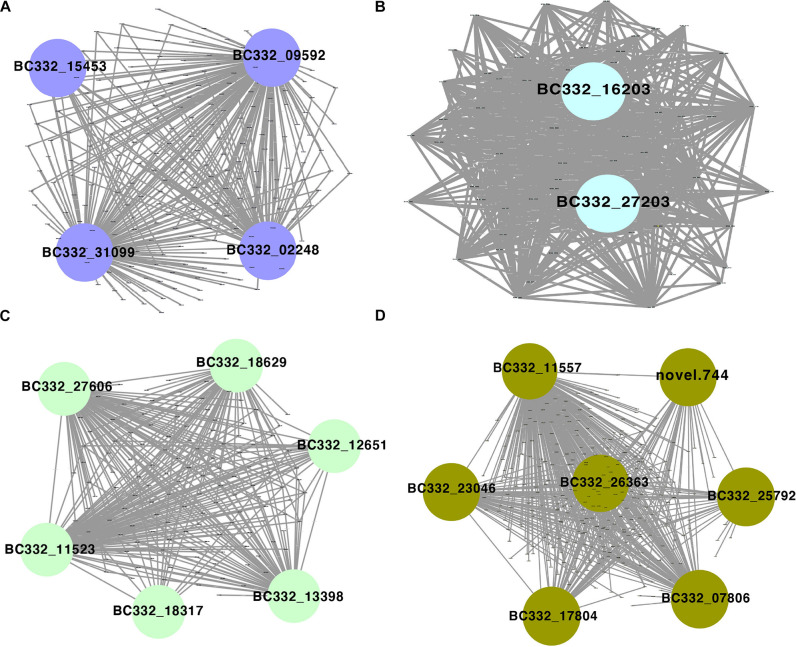
Gene networks and hub genes involved in heterosis regulation during pepper development by WGCNA analysis. **(A)** Gene network of the purple module. **(B)** Gene network of the lightsheetblue1 module. **(C)** Gene network of the white module. **(D)** Gene network of the black module.

Among the 42 coexpressed gene modules, each expression cluster was displayed in a heatmap, which can directly visualize the relationship between the clusters of pepper at two development stages (Figure 5B). Subsequently, eight horticulture phenotypic data of module–trait correlations were analyzed at the two developmental stages. We found that physiological indexes were mainly concentrated in purple at LA and plant stem diameter (PSD) with a moderate correlation coefficient of 0.45–0.56 (*p* < 0.05). Besides, the ligthsteelblue1 module displayed a positive correlation with plant scope (PS), FL, and main stem height (MSH), while the black and white modules were significantly positively correlated with PH and FW at F-stage. Furthermore, the genes with a higher weight in each module were chosen for network constructing and analysis (Figure 6). By searching the hub gene of the major gene network, *BC332_ 11557*, *novel.744*, *BC332_18317*, and *BC332_23046* were related to β-glucosidase and auxin responsive (Table 3). These results exhibited that these genes may have a relationship with the heterosis of pepper.

**TABLE 3:** The detailed information of hub genes.

Gene ID	Module	Description	Pattern
BC332_31099	Purple	Putative protein phosphatase 2C 53	Over-dominance
BC332_09592	Purple	Protein phosphatase 2C 16	Over-dominance
BC332_02248	Purple	Transcription factor bHLH80	Over-dominance
BC332_15453	Purple	Heat stress transcription factor A-2e	Over-dominance
BC332_27606	White	Ethylene-responsive transcription factor WIN1	Over-dominance
BC332_13398	White	Ethylene-responsive transcription factor	Over-dominance
BC332_11523	White	Serine/threonine-protein kinase SAPK2	Over-dominance
BC332_18629	White	Transcription factor PIF1	Over-dominance
BC332_12651	White	Transcription factor TGA7	Over-dominance
BC332_18317	White	Auxin-responsive protein IAA20	Over-dominance
BC332_07806	Black	Flowering locus K -like proteiny domain	Over-dominance
BC332_17804	Black	Transcription factor bHLH66	Over-dominance
BC332_23046	Black	Auxin response factor 8	Over-dominance
BC332_25792	Black	Transcription factor RF2b	High-parental dominance
BC332_26363	Black	Serine/threonine-protein kinase CTR1	Over-dominance
BC332_11557	Black	Beta-1%2C3-galactosyltransferase 7	Over-dominance
novel.744	Black	Beta-glucosidase 9	Over-dominance
BC332_27203	Lightsheetblue1	Putative receptor-like protein kinase	Over-dominance
BC332_16203	Lightsheetblue1	ATP-dependent zinc metalloprotease FtsH 3	N/A

### Analysis of DE lncRNAs, DE miRNAs and DE circ RNAs

**FIGURE 7:**
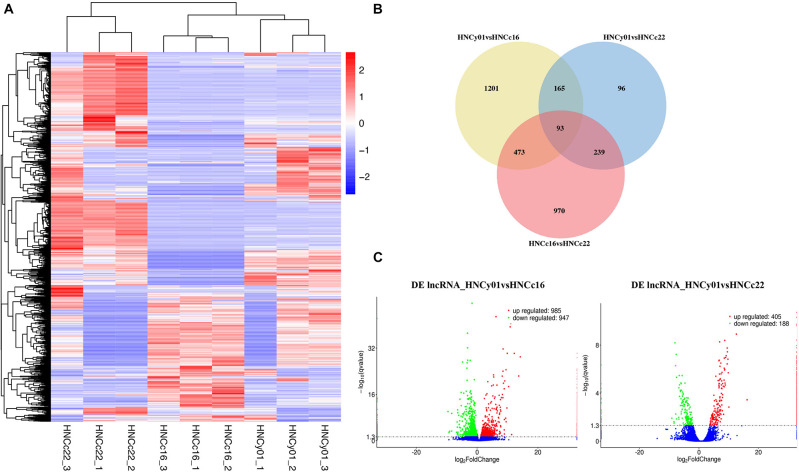
Significantly differentially expressed lncRNAs between the hybrid and its parents at the seedling stage. **(A)** Hierarchical cluster analysis of DE lncRNAs. **(B)** Venn diagram of DE lncRNAs in HNCy01vsHNCc16, HNCy01vs HNCc22, and HNCc16vs HNCc22. **(C)** Volcano plot of DE lncRNAs in HNCy01vsHNCc16 and HNCy0vsHNCc22. Upregulated, downregulated, and non-differentially expressed genes are represented by red, green, and blue dots, respectively.

The heat map illustrated that the F_1_ was more similar to its parental line HNCc16 (Figure 7A). Venn diagram showed that 1,201 and 96 genes had a unique expression in HNCy01vs HNCc16 and HNCy01vs HNCc22. Moreover, 93 DE lncRNAs were commonly expressed among all three genotypes (Figure 7B). In addition, we identified 1,932 DE lncRNAs (985 upregulated and 947 downregulated) and 593 DE lncRNAs (405 upregulated and 188 downregulated) in HNCy01 compared with HNCc16 and HNCc22 at S-stage, respectively (Figure 7C and Supplementary Table 5).

A total of 17 DE miRNAs (seven upregulated and 10 downregulated) were identified in HNCy01and HNCc16, and 30 DE miRNAs (15 upregulated and 15 downregulated) were identified in HNCy01 and HNCc22. These DE miRNAs include 13 known miRNA and 34 novel miRNAs, of which conserved miRNAs belong to seven families (Supplementary Figure 4 and Supplementary Table 6). There were 54 DE circRNAs (27 upregulated and 27 downregulated) in HNCy01 compared to HNCc16. In comparison with HNCc22, we identified 17 DE circRNAs (two upregulated and 15 downregulated) in HNCy01 (Supplementary Figure 5 and Supplementary Table 7).

**FIGURE 8:**
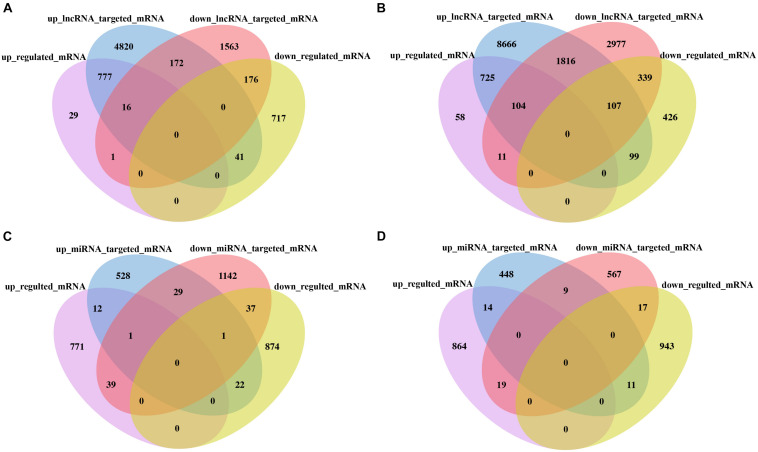
Analysis of the targeted mRNA of lncRNA and miRNA between the hybrid and its parents. **(A)** Venn diagram of DE lncRNA targeted mRNA between HNCy01 and HNCc22. **(B)** Venn diagram of DE lncRNA targeted mRNA between HNCy01 and HNCc16. **(C)** Venn diagram of DE miRNA targeted mRNA between HNCy01 and HNCc22. **(D)** Venn diagram of DE miRNA targeted mRNA between HNCy01 and HNCc16.

To better reveal the DE lncRNA and DE miRNA functions, through analysis of targeted mRNA of DE lncRNA and DE miRNA, we identified 777 (725) upregulated targeted mRNA of upregulated lncRNA and 176 (339) downregulated targeted mRNA of downregulated lncRNAs in the F_1_ hybrid in comparison with its parent HNCc22 (HNCc16). Similarity, one (11) upregulated targeted mRNA of downregulated lncRNAs and 41 (99) downregulated targeted mRNAs of upregulated lncRNAs in the F_1_ hybrid in comparison with its parent HNCc22 (HNCc16) were identified (Figures 8A,B and Supplementary Table 8). Furthermore, by intersection analysis of DE miRNAs, we found 39 (19) upregulated targeted mRNAs of downregulated miRNAs and 22 (11) downregulated targeted mRNAs of upregulated miRNAs in the F_1_ hybrid compared with its parent HNCc22 (HNCc16) (Figures 8C,D and Supplementary Table 9). We also identified one upregulated targeted mRNA from one upregulated circRNA and two downregulated targeted mRNAs from two downregulated circRNAs (Table 4).

**TABLE 4:** The detailed information of DE circRNA and its targeted mRNA.

circRNA ID	circRNA regulation	DE targeted mRNA ID	mRNA regulation	Description
novel_circ_0001361	Up	BC332_22932	Up	Probable aminotransferase
novel_circ_0000872	Down	BC332_16958	Down	Chloroplast stem-loop binding protein
novel_circ_0001206	Down	BC332_22540	Down	Kinesin-like protein

By GO enrichment analysis of the targeted mRNA of DE lncRNAs, GO terms binding (GO:0005488) was remarkably enriched in molecular function categories. Among the biological process categories, the signal–organism process (GO:0044699) was highly represented. Membrane (GO:0016020) and membrane part (GO:0044425) were dominated in cellular components. GO enrichment of the targeted mRNA of DE miRNAs showed that binding (GO:0005488) was enriched in molecular function categories. In addition, phosphorylation (GO:0016310) and protein phosphorylation (GO:0006468) were significantly enriched in biological process categories. KEGG enrichment analysis of DE lncRNAs suggested that photosynthesis and metabolic pathways were enriched (Supplementary Figure 6).

**FIGURE 9:**
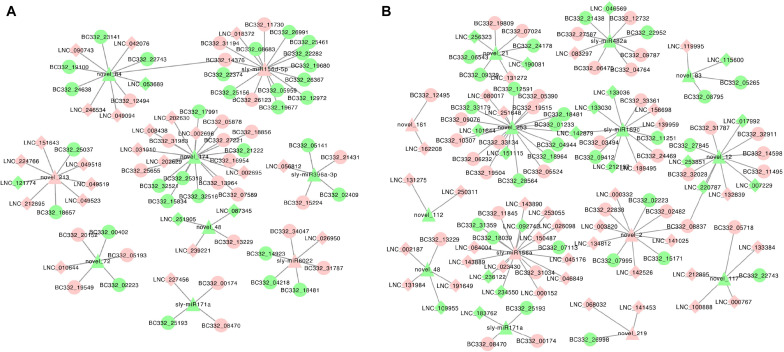
The visualization of coexpression network in the hybrid and its parents. **(A)** lncRNA–miRNA–mRNA coexpression network between HNCy01 and HNCc22. **(B)** lncRNA–miRNA–mRNA coexpression network in HNCy01 vs. HNCc16. Red indicates upregulated, green indicates downregulated, ellipse indicates mRNA, diamond indicates lncRNA, and triangle indicates miRNA, respectively.

We constructed the lncRNA–miRNA–mRNA coexpression network. In HNCy01 and HNCc22, 249 lncRNA–miRNA–mRNA combinations were identified, including 26 lncRNAs as decoys, nine miRNAs as centers, and 70 mRNAs as targets. HNCy01 vs. HNCc16 showed 293 lncRNA–miRNA–mRNA combinations with 55 lncRNAs, 14 miRNAs, and 60 mRNAs (Figure 9 and Supplementary Table 10).

### DEGs Encoding Transcription Factor

A total of 2,145 differentially expressed TFs were identified at the two developmental stages and belonged to 57 TF families, including 213 bHLH, 176 MYB-related, 128 ERF, 118 WRKY, 116 NAC, 110 B3, 89 C3H, 81 MYB, 76 M-type MADS, and 39 HSF. Among these TFs, the bHLH family accounted for the largest proportion, followed by MYB-related family ERF family protein and WRKY family at two developmental stages. Furthermore, 32.17% (690/2,145) TFs showed high-parental dominance or over-dominance, which indicated that these TFs might play an important function in pepper heterosis (Supplementary Table 11).

## Discussion

Heterosis utilization plays a crucial role in crop production. Our current knowledge of heterosis molecular mechanism is mainly based on rice and *Arabidopsis*. However, the understanding of the pepper molecular mechanism heterosis is still limited. Here, we used non-directional sequencing, strand-specific RNA sequencing, and miRNA analysis to understand the heterosis of pepper hybrid at two development stages, providing important preliminary data and clues for the further study of heterosis in pepper.

### Phenotype Change in the F_1_ Hybrid

The leaf photosynthesis is crucial for plant development (Yang et al., 2018). A recent study has proved that the *Arabidopsis* F_1_ hybrid germinated earlier and has a larger leaf size than its parents. Furthermore, the larger size of the leaf was associated with increased cell numbers with a greater number of chloroplasts, which increased photosynthate production contributing to higher biomass (Fujimoto et al., 2012; Groszmann et al., 2014; Liu et al., 2020a). In our study, the pepper F_1_ hybrid has a larger LA and grows quicker than its parents. Besides, compared with its parents, the hybrid HNCy01 has the over-dominance advantage in crown diameter. However, the physiological basis of these advantages needs further analysis.

### Non-additive Effect Role in Heterosis

Using RNA-seq analysis, our comparative transcriptome analysis revealed a subset of differential expression transcripts between the F_1_ hybrid and its parents at the S-stage and F-stage. Several reports suggested that a great number of genes showed additive expression patterns in hybrids, with only a small proportion of genes having non-additive expression patterns (Hu et al., 2016; Shen et al., 2017), while it has also been reported that non-additive genes play a vital role in gene expression and heterosis (Zhang et al., 2008; Wei et al., 2009; Schnable and Springer, 2013; Yang et al., 2018). In this study, we have investigated that a majority proportion of differential expressed genes possessed a non-additive pattern in the F_1_ hybrid from HNCc22 and HNCc16, suggesting that dominance models could be associated with growth vigor (Hedgecock et al., 2007).

### Coexpression Facilitates the Identification of Traits-Related Key Genes

The weighted gene coexpression network analysis revealed the module hub genes related to the eigengene traits of pepper at two developmental stages. Here, we found that several modules have a significantly positive correlation between LA and seedling stem diameter. In the purple module, we found that β-glucosidase was highly expressed, supported by a recent report that β-glucosidase plays a crucial role in stomatal traits and photosynthesis (Zhou et al., 2021). We found that *BC332_18317* and *BC332_23046* were over-dominance expressed in the white and black modules, encoding auxin-responsive protein. KEGG pathway analysis also identified these genes in plant hormone regulation, indicating their possible roles in plant morphology construction and pepper growth at a specific developmental stage.

### Plant Hormone Signal Transduction Contributing to Heterosis in Hybrid

In multicellular organisms, hormones have been shown to coordinate with cell division, expansion, differentiation, and stress (Malamy et al., 1990; Tan et al., 2007; Santiago et al., 2009; Kyndt et al., 2012; Davière and Achard, 2013; Zhai et al., 2013; Lavy and Estelle, 2016; Huang et al., 2017; Shani et al., 2017; Ding et al., 2018; Kunkel and Harper, 2018; Luo et al., 2018; Wang et al., 2020). This study found that many DEGs were over-dominant and related to plant hormone signal transduction including auxin, gibberellin, abscisic acid (ABA), ethylene, jasmonic acid, and salicylic acid. For example, members from ABA signal transduction pathway, *PYR/PYL* (ABA receptor group), were upregulated at S-stage and downregulated at F-stage, and PP2C were upregulated in the F_1_ hybrid (Santiago et al., 2009), which showed the negative-feedback regulatory mechanism of ABA signal transduction, similar to the previous study on rice root heterosis (Zhai et al., 2013). TGAs, positive regulators of SA-induced PR genes (Ding et al., 2018), were upregulated in the F_1_ hybrid in the salicylic acid pathway. And NPR protein as an SA receptor was upregulated at F-stage, suggesting that the hybrid may regulate plant immunity based on heterosis (Wang et al., 2020). In auxin signaling pathway, AUX/IAA and SAUR controlling plant growth by cell enlargement were upregulated in the F_1_ hybrid. Moreover, previous research reported that the gibberellin (GA) signaling pathway gives impetus to the transformation of vegetative into reproduction growth and stress tolerance (Colebrook et al., 2014). DELLA proteins restrain plant growth and GID1 as a GA receptor. In the present study, GID1 was upregulated at the S-stage but downregulated at the F-stage, which indicates that GA signal promotes growth by overcoming DELLA-mediated growth restraint (Davière and Achard, 2013). We also identified that *BC332_21619* and *BC332_25121* belong to the bHLH family, which were over-dominant in both the S-stage and the F-stage. These results will be helpful for future studies on molecular mechanisms of hormones underlying pepper heterosis.

### miRNA Roles in Heterosis

The miRNA is a kind of small RNA, which plays an important role in gene expression, defense responses, and cell function regulation in plants and animals (Zhang et al., 2013). Recent studies showed that several miRNAs showed non-additive expression and led to the non-additive expression of target genes affecting growth vigor and adaptability (Ng et al., 2012; Chen, 2013). Here, we also found plenty of DE miRNAs and their target DE mRNAs in the F_1_ hybrid. It has been reported that miR156 may participate in flowering and abiotic stress through targeted gene regulation (Wang et al., 2009; Frazier et al., 2011; Khraiwesh et al., 2012). For example, the upregulation of miR156 may be beneficial for higher anthocyanin synthesis under drought stress (González-Villagra et al., 2017). Besides, it was also found that *GmmiR156b* might improve shoot architecture and yield in soybean (Sun et al., 2019; Liu et al., 2020b). We found that *miR156a* and *miR156d-5p* expressions were high-parental dominant in the pepper F_1_ hybrid HNCy01. The miR169 family has been reported to be associated with ABA-responsive TFs (Ding et al., 2012; Song et al., 2018). In the allopolyploid wheat, *miR169.2* and *miR169.6* showed an expression pattern with low-parental ELD-ab. Similarly, in our pepper hybrid, miR169c was downregulated compared with both parents. The miR171 family was known to regulate chlorophyll biosynthesis and leaf growth by target TFs DELLA (Ma et al., 2014). The miR369 was reported to be involved in growth-regulating factors (GRF) silence, which may negatively regulate in disease resistance (Chandran et al., 2018). The other research reported that the overexpression of miR369 has a negative influence on PH, which may be associated with biomass yield (Liu et al., 2021), while in our study, miR369a-3p and miR369a-5p were downregulated in HNCy01 and HNCc22, which may have advantageous impacts on pepper growth. Based on these results, it is suggested that miRNA could play an important role in pepper heterosis.

### LncRNA Function in Heterosis

Plant lncRNAs have strong relationships with abiotic and biotic responses, photomorphogenesis, flowering time regulation, etc. (Wang H. et al., 2014, Wang Y. et al., 2014, Wang Z.W. et al., 2014, Wang et al., 2015). In this study, we identified a great number of lncRNAs by strand-specific RNA-seq. In the F_1_ hybrid HNCy01, more upregulated lncRNAs were enriched compared to downregulated lncRNAs. Meanwhile, the target mRNAs of many DE lncRNAs were also differentially expressed, suggesting their roles in pepper heterosis. Furthermore, we identified 74 upregulated lncRNAs in the plant–pathogen interaction pathway from HNCy01 vs. HNCc16. However, the functions of some plant lncRNAs, such as COOLAIR mediating epigenetic silence of the floral repressor FLOWERING LOCUS C, were described in detail (Zhao et al., 2018). Although large-scale lncRNAs were identified, it is still a challenge to reveal the function and mechanism of lncRNA, and the roles of lncRNAs in plant heterosis remain to be further clarified.

### Transcription Factors Regulate the Heterosis of Pepper

Transcription factors, a group of DNA-binding proteins controlling gene transcription, play crucial roles in heterosis (Zhang et al., 2008; Guo et al., 2017; Chen et al., 2018). Numerous TFs have been reported to be correlated with plant development and resistance ability. For example, MYB TFs play a crucial role in various biological processes. Previous research demonstrates that the MYB family functioned in plant growth and development and even abiotic stress (Wang et al., 2011). bZIP TFs are involved in PH and flower development (Jakoby et al., 2002).

Furthermore, among these TF families, 690 TF genes were displaying high-parental dominance or over-dominance. ARF family genes, which are responsive to plant hormone transduction, were highly expressed in shoots (Liu et al., 2014). We found that ARF family genes had over-dominance expression at the S-stage and high-parental dominance expression at the F-stage. Besides, we identified 39 HSF TFs implementing important functions in response to high-temperature stress and plant development (Guo et al., 2015). In our study, 22 TFs displayed over-dominance or high-parental dominance, suggesting that these TFs may play a crucial factor in resistance ability.

The bHLH family plays a significant role in phytohormone signal and abiotic stress (Zhou et al., 2021). MYB TFs are key factors in cell proliferation and differentiation (Dubos et al., 2010). For the network analysis, we identified that bHLH and MYB TFs were overdominant in the white and black module, suggesting that these genes may have a relationship with pepper development, indicating their potential roles in heterosis (Ni et al., 2009).

## Conclusion

This study used RNA-seq analysis to investigate the transcriptome of pepper hybrid and their parents at the S-stage and F-stage. We identified plenty of DEGs and novel ncRNAs in three genotypes. Comparing the significantly enriched gene between the F_1_ hybrid and its parents, we have identified some candidate transcripts that may correlate with heterosis. This study will provide molecular resources for further interpreting the pepper heterosis mechanism.

## Data Availability Statement

The datasets presented in this study can be found in online repositories. The names of the repository/repositories and accession number(s) can be found in the article/Supplementary Material.

## Author Contributions

Z-WW and H-YS: conceptualization and methodology. H-YS: software and writing—original draft preparation. H-YS, HZ, and H-LM: validation. H-YS, HZ, H-LM, Y-LJ, ZY, Y-YH, JZ, W-LB, S-HC, and G-PZ: investigation. Z-WW, H-YS, H-LM, Y-LJ, ZY, Y-YH, JZ, W-LB, S-HC, and G-PZ: writing—review and editing. Z-WW: supervision. All authors read and agreed to the published version of the manuscript.

## Conflict of Interest

The authors declare that the research was conducted in the absence of any commercial or financial relationships that could be construed as a potential conflict of interest.

## Publisher’s Note

All claims expressed in this article are solely those of the authors and do not necessarily represent those of their affiliated organizations, or those of the publisher, the editors and the reviewers. Any product that may be evaluated in this article, or claim that may be made by its manufacturer, is not guaranteed or endorsed by the publisher.

## Footnotes

1
https://www.ncbi.nlm.nih.gov/genome/?term=Capsicum+chinense

2
https://trace.ncbi.nlm.nih.gov/Traces/sra/sra.cgi?

3
https://cytoscape.org/

4
http://jvenn.toulouse.inra.fr/app/usermanual.html

5
http://planttfdb.gao-lab.org/
