# Supplementary material for: Integrated Analysis of mRNA and Non-coding RNA Transcriptome in Pepper (Capsicum chinense) Hybrid at Seedling and Flowering Stages
Source: Front Genet. 2021 Aug 20;12:685788. doi: 10.3389/fgene.2021.685788 (PMC8417703; doi:10.3389/fgene.2021.685788)
Supplement: Supplementary file 1 [file Table_1.DOCX]

Supplementary Material

**Supplementary Figure 1.** The number of DEGs of up-regulated and down regulated at S-stage and F-stage.

** Supplementary Figure 2.** KEGG pathway assignments of DEGs. (A) KEGG analysis at S-stage; (B) KEGG analysis at F-stage.

**Supplementary Figure 3.** Cluster dendrogram and network heatmap plot of selected genes to coexpression module.


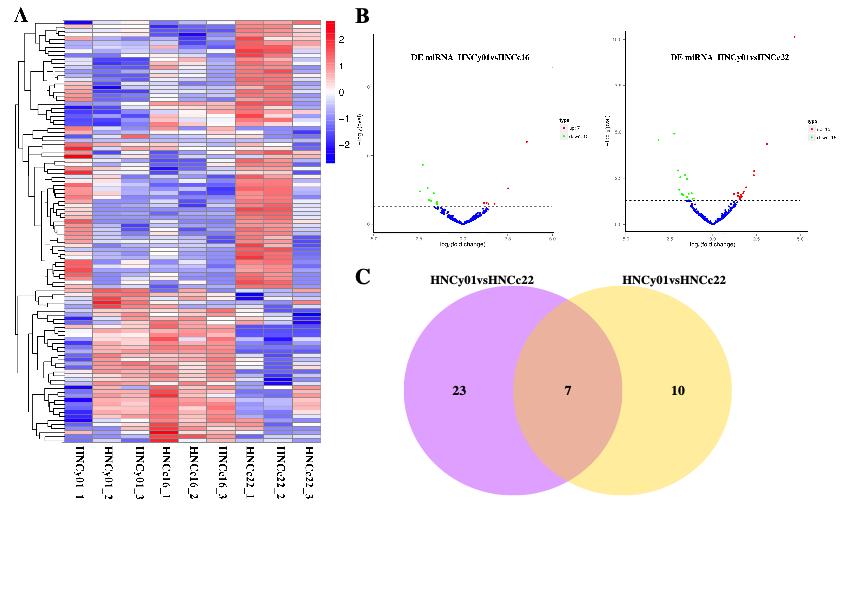


**Supplementary Figure 4.** Significantly differentially expressed miRNAs between the hybrid and its parents. (A) Hierarchical cluster analysis of DE miRNAs. (B)Volcano plot of DE miRNAs between HNCy01vs HNCc16 and HNCy01vs HNCc22. Up-regulated, down-regulated and non-differentially expressed genes were represented by red, green and blue dots, respectively. (C) Venn diagram of the number of DE miRNAs between HNCy01vsHNCc16 and HNCy01vsHNCc22.

** Supplementary Figure 5.** Significantly differentially expressed circRNAs between the hybrid and its parents. (A) Hierarchical cluster analysis of DE circRNAs. (B)Volcano plot of DE circRNAs between HNCy01vs HNCc16 and HNCy01vs HNCc22. Up-regulated, down-regulated and non-differentially expressed genes were represented by red, green and blue dots, respectively. (C) Venn diagram of the number of DE circRNAs between HNCy01vsHNCc16 and HNCy01vsHNCc22.

*
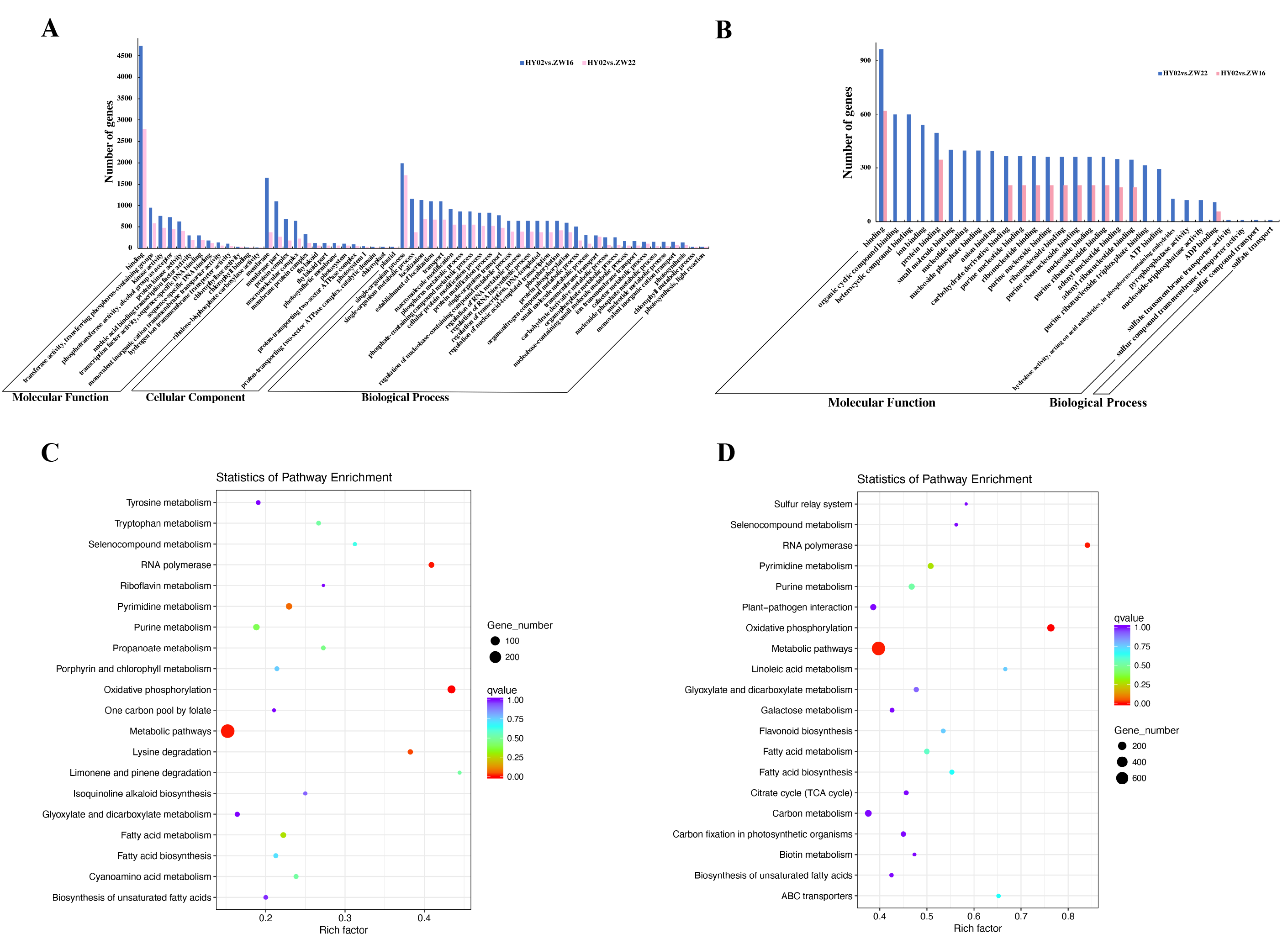
*

**Supplementary Figure 6.** Enrichment analysis of DE lncRNA and DE miRNA between the hybrid and its parents. (A) GO analysis of DE lncRNAs between HNCy01 vs. HNCc16 and HNCy01 vs. HNCc22. (B)Analysis of DE miRNAs in HNCy01 vs. HNCc16 and HNCy01 vs. HNCc22, and (C) KEGG analysis of DE lncRNAs in HNCy01 vs. HNCc22. (D) KEGG analysis of DE lncRNAs in HNCy01 vs. HNCc16.
